# Optimisation of Ultrasound Frequency, Extraction Time and Solvent for the Recovery of Polyphenols, Phlorotannins and Associated Antioxidant Activity from Brown Seaweeds

**DOI:** 10.3390/md18050250

**Published:** 2020-05-11

**Authors:** Viruja Ummat, Brijesh K Tiwari, Amit K Jaiswal, Kevin Condon, Marco Garcia-Vaquero, John O’Doherty, Colm O’Donnell, Gaurav Rajauria

**Affiliations:** 1 School of Biosystems and Food Engineering, University College Dublin, Belfield, D04 V1W8 Dublin, Ireland; viruja.ummat@ucdconnect.ie (V.U.); colm.odonnell@ucd.ie (C.O.); 2 Teagasc Food Research Centre, Ashtown, D15 DY05 Dublin, Ireland; brijesh.tiwari@teagasc.ie; 3 School of Food Science and Environmental Health, College of Sciences and Health, Technological University Dublin—City Campus, Grangegorman, D07 EWV4 Dublin, Ireland; amit.jaiswal@tudublin.ie (A.K.J.); C15467412@mytudublin.ie (K.C.); 4 School of Agriculture and Food Science, University College Dublin, Belfield, D04 V1W8 Dublin, Ireland; marco.garciavaquero@ucd.ie (M.G.-V.); john.vodoherty@ucd.ie (J.O.)

**Keywords:** ultrasound assisted extraction, conventional extraction, polyphenols, phlorotannin, macroalgae, antioxidant capacity

## Abstract

This study investigates ultrasound assisted extraction (UAE) process parameters (time, frequency and solvent) to obtain high yields of phlorotannins, flavonoids, total phenolics and associated antioxidant activities from 11 brown seaweed species. Optimised UAE conditions (35 kHz, 30 min and 50% ethanol) significantly improved the extraction yield from 1.5-fold to 2.2-fold in all seaweeds investigated compared to solvent extraction. Using ultrasound, the highest recovery of total phenolics (TPC: 572.3 ± 3.2 mg gallic acid equivalent/g), total phlorotannins (TPhC: 476.3 ± 2.2 mg phloroglucinol equivalent/g) and total flavonoids (TFC: 281.0 ± 1.7 mg quercetin equivalent/g) was obtained from *Fucus vesiculosus* seaweed. While the lowest recovery of TPC (72.6 ± 2.9 mg GAE/g), TPhC (50.3 ± 2.0 mg PGE/g) and TFC (15.2 ± 3.3 mg QE/g) was obtained from *Laminaria digitata* seaweed. However, extracts from *Fucus serratus* obtained by UAE exhibited the strongest 1,1-diphenyl-2-picryl-hydrazyl (DPPH) scavenging activity (29.1 ± 0.25 mg trolox equivalent/g) and ferric reducing antioxidant power (FRAP) value (63.9 ± 0.74 mg trolox equivalent/g). UAE under optimised conditions was an effective, low-cost and eco-friendly technique to recover biologically active polyphenols from 11 brown seaweed species.

## 1. Introduction

The consumption of seaweeds is a long tradition in many Asian countries and recently has also increased in Europe and North America [1]. Many seaweed species contain significant quantities of compounds such as polyphenols, polysaccharides, carotenoids, fibres, minerals, trace elements, proteins and amino acids [2,3,4]. Moreover, brown seaweeds are good sources of polyphenolic compounds such as phlorotannins, flavanols and catechins [5]. Phlorotannins are a specific group of polyphenols produced by brown seaweeds that have gained recognition for their broad range of potential biological properties which are beneficial to humans [5,6,7]. The potential beneficial biological properties of polyphenols include antioxidant, antimicrobial, antiviral, anticancer, anti-inflammatory and antidiabetic activities [8]. These phenolic compounds are not only being explored for their biological activities but also for their potential to prevent several chronic diseases such as cancer, cardiovascular diseases, obesity and diabetes [9]. Due to their nutritional and health benefits, algal polyphenols are increasingly being investigated for their possible use in nutraceuticals, functional foods, cosmetic, and pharmaceutical applications [10]. 

The increasing interest in utilising brown seaweeds as a sustainable biosource material for bioactive recovery has fuelled the development of new extraction/pre-treatment technologies. *Fucus vesiculosus* or bladderwrack is rich in sulphated polysaccharides (such as fucoidan) as well as polyphenols (such as phlorotannins). The species is reported to have the highest phlorotannin (6% DW) and fucoidan (up to 20% DW) contents among *Fucus* species [2]. However, it has been mainly investigated to produce fucoidan, while its high phenolic content including phlorotannins has not been exploited to date. The recovery of polysaccharide is mainly carried out using acidic water as a solvent, while its phenolic content is either unextracted and stays in the residue or removed during purification, if co-extracted. Extraction of polyphenolic compounds is a huge challenge as they are embedded deeply within the seaweed matrix. Traditional polyphenol extraction from seaweeds has relied on methods that require high energy consumption, long extraction periods, low yields and the use of potentially toxic chemical agents and solvents [11]. A range of solvents both chlorinated (chloroform, chlorobenzene, carbon tetrachloride, tetrachloroethylene) and non-chlorinated (methanol, ethanol, acetone) are used for extraction of these compounds. However, toxicity, low yield of extracts and prevalence of residues in the target compound have always been a concern. Extraction of these compounds at a commercial scale requires high extraction yields as well as intact biological activities, which are difficult to achieve using conventional extraction methods [4]. Therefore, to address these shortcomings, and to facilitate the transition towards more environmentally sustainable extraction technologies, there is a need to develop new, safe, effective and affordable extraction technologies, which give maximum product yield, minimum presence of residues and enable attainment of clean label status. Recently, several greener and cleaner extraction technologies were investigated which have greater extraction efficiency and lower carbon footprints [12]. 

Novel extraction technologies also referred to as cold extraction techniques such as ultrasound, due to the comparatively low temperatures employed during the process, have minimal impact on the stability of the target compounds, have high potential to reduce or eliminate the use of toxic chemicals, increase process efficiency and enhance yield and quality of the target products [13,14]. Ultrasound technology can be used either as a pre-treatment or in combination with other safe organic solvents to disrupt cell membranes to enable better extractability [13]. Thus, the motive to develop a sustainable extraction process has led to more research on ultrasound assisted extraction (UAE), owing to decreased levels of solvent consumption, shorter duration of extraction and reduced operational costs [15]. Ultrasound involves various physical and chemical phenomena including compression rarefaction, vibration, pressure, shear forces, microjets, agitation, cavitation and radical formation. However, the main driving force for extraction is acoustic cavitation, which involves creation, expansion and implosive collapse of micro bubbles, formed due to a series of compression and rarefactions in molecules, generated by ultrasound waves [13]. The ability of ultrasound to cause cavitation, for extraction and processing applications, depends on UAE process parameters such as frequency and sonication intensity within the range of 10–1000 W/cm^2^. Other process parameters which influence extraction include time, temperature, pressure applied during the process and properties of the extraction solvent (e.g., viscosity and surface tension) [13]. Several methods for extraction of phenolic content from seaweed have been investigated, however, limited data are available on the effect of UAE conditions to extract phenolic compounds from a range of seaweeds and the resultant biological properties of the macroalgal extracts. Therefore, this study aims to (1) optimise UAE process parameters (time, frequency, solvent ) to obtain high extraction yields and recovery of phlorotannins and total phenols from *F. vesiculosus* (2) assess the damage caused by ultrasound on algal cell surfaces using scanning electron microscopic (SEM) analysis and (3) investigate the extraction efficiency of optimised UAE conditions and conventional solvent extraction techniques to obtain phenolic constituents (phlorotannins, flavonoids and total phenolic compounds) from 11 brown macroalgae species widely available in Ireland. 

## 2. Results and Discussion

### 2.1. Effect of Ultrasound on Extraction Yield, Total Polyphenols and Phlorotannin Content

**Figure 1:**
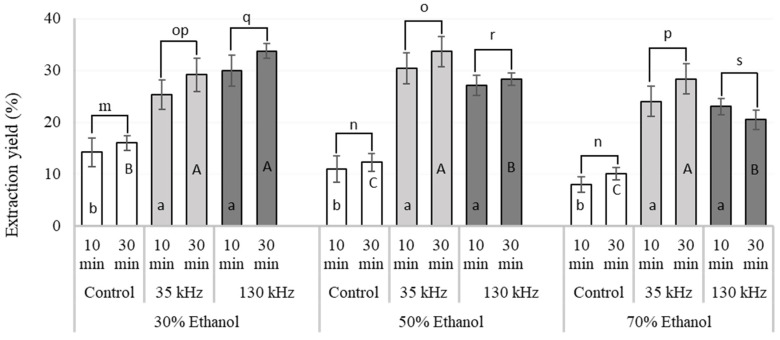
Effects of ultrasound assisted extraction (UAE) conditions (solvent concentration (30%, 50% and 70% ethanol), ultrasonic frequency (control, 35 kHz and 130 kHz) and UAE treatment time (10 and 30 min)) on the extract yield obtained from *F. vesiculosus*. Different letters indicate statistical differences (*p* < 0.05) on the yields of seaweed extract obtained using different solvents at each US frequency: control (m-n), 35 kHz (o-p) and 130 kHz (q-s). **^abc^** columns with similar letters are not significantly different (*p* < 0.05) treated for 10 min; ABC columns with similar letters are not significantly different (*p* < 0.05) treated for 30 min. The extraction yield is calculated by using following formula: Extraction yield (%) = (weight of dry extract / weight of dry sample) × 100.

*F. vesiculosus* brown seaweed was used as the raw material for optimisation of UAE conditions (time, frequency and solvent) to obtain high recovery of total polyphenol and phlorotannin content. Figure 1 shows the effects of ethanol concentration (30%, 50% and 70% v/v), ultrasound frequency (0 kHz or control, 35 kHz and 130 kHz) and UAE treatment time (10 and 30 min) on extraction yield (%). There was a statistically significant interaction observed between ultrasonic frequency and solvent type (*p* < 0.001). No statistical differences were observed within each extraction treatment by applying these conditions for 10 or 30 min. Further statistical analyses were performed to elucidate the effects of different solvents at each US frequency on the yield of extracts as shown in Figure 1. Irrespective of extraction solvents, application of ultrasound significantly improved the extraction yield from 78.7% to 201.8% with respect to the controls. Considerable variations in extraction yield were observed between controls and treatments. Among the controls, the highest extraction yield (16%) was recorded for 30% ethanol, while the lowest yield (8%) was recorded from 70% ethanol. Interestingly, the yield started to decrease as the concentration of ethanol increased which is in agreement with previously reported findings [16,17]. Among the UAE treatments investigated, both the highest (33.8%) and the lowest (20.5%) extraction yields were observed at 130 kHz US frequency and 30 min extraction time but at different ethanol concentrations (30% and 70%, respectively) (Figure 1). Therefore, ultrasonic frequency of 35 kHz which is less energy intensive and provides similar extraction yields for the same treatment time, was considered as the best frequency to recover high yields of extract from *F. vesiculosus*. 

Similar findings were reported in other studies using other innovative extraction technologies. He et al. [18] observed that phlorotannin yields from *Saccharina japonica* increased with ethanol concentration over the range of 40–50% for microwave-assisted extraction. The same authors reported a reduction in the yields of compounds extracted when using ethanol concentrations above 50% due to an increased extraction of other less polar components. Another study on optimisation of the extraction variables on the yields of crude extracts from *Ascophyllum nodosum*, also observed that ethanol concentration had a significant effect on the yield of extract obtained, and that lower ethanol concentrations enhanced the extraction yield, suggesting that most of the extractable compounds were high in polarity [19].

Table 1 summarises the recovery of total polyphenols and phlorotannins extracted as well as retained (unextracted) in the residue of *F. vesiculosus* seaweed after ultrasound treatment. There was a statistically significant (*p* < 0.001) influence of the interaction solvent × ultrasonic frequency × extraction time on the recovery of bioactive compounds. The influence of the extraction time (10 and 30 min) on the recovery of TPC and TPhC for each extraction condition is shown in Table 1. Extraction using 50% ethanol yielded more polyphenols and phlorotannins in control samples while 30% ethanol generated more in treated samples, irrespective of US treatments frequencies. As shown in Table 1, values of TPC and TPhC obtained in treated samples were in the range of 422.7–579.7 mg GAE/g and 327.2–471.5 mg PGE/g, respectively, of dried extract, while the control samples exhibited values in the range of 306.8–358.5 mg GAE/g and 222–286.2 mg PGE/g, respectively. Among the treatments, samples treated with 35 kHz US frequency in 30% ethanol for 10 min yielded the lowest amount of TPC (422.7 ± 4.4mg/g) and TPhC (327.2 ± 7.2 mg/g). Samples treated with 130 kHz US frequency in 30% ethanol for 30 min yielded the highest amount of TPC (579.7 ± 9.2 mg/g) and TPhC (471.5 ± 7.5 mg/g), however these values were statistically similar (*p* > 0.05) with the values obtained from the samples treated with 35 kHz US frequency in 50% ethanol for 30 min (TPC: 571.1 ± 10.0 mg/g and TPhC: 462.6 ± 2.1 mg/g) (Table 1). As 35 kHz US frequency utilises less energy compared to 130 kHz in the same extraction time (30 min) and yields were statistically similar for TPC and TPhC values, 35 kHz US frequency, 50% ethanol and 30 min were considered the optimum conditions for recovery. Interestingly, TPhC contributed 70.6–87.9% to both the lowest and highest TPC amounts. Moreover, control samples exhibited a strong solvent and time effect. All the control samples showed lower TPC values at 10 min treatment time compared to 30 min treatment time. Control samples extracted with 50% ethanol contained the highest TPC at 30 min, while the lowest TPC content was observed for 70% ethanol at 10 min. Compared to the control, ultrasound treatment significantly improved the extraction efficiency of TPC and TPhC, irrespective of treatment time and solvent used. The extraction efficiency calculated by using Equation (1) (Equation (1)) revealed that the highest extraction efficiency (70.3%) of TPC was achieved at 130 kHz for 30 min in 30% ethanol, while the lowest efficiency (32.2%) was observed at 130 kHz for 30 min in 50% ethanol (Table 1). The highest extraction efficiency (89.6%) of TPhC was achieved at 130 kHz in 30% ethanol for 30 min, while the lowest efficiency (28.1%) was observed at 130 kHz for 30 min in 50% ethanol. Though the extraction efficiencies for TPC and TPhC were higher at 130 kHz for 30 min in 30% ethanol compared to 35 kHz for 30 min in 50% ethanol (selected optimum conditions), extraction at 35 kHz is less energy intensive and yielded statistically similar (*p* > 0.05) TPC and TPhC to 130 kHz (Table 1), and was thus considered the best condition for extraction. Similar results were obtained in a study by Kadam et al. [20], in which the effects of ultrasound amplitude (22.8–114 µm), extraction time (5–25 min) and acid concentration (0–0.06 M HCl) on total phenolics, fucose and uronic acids from *A. nodosum* were investigated. The authors reported that the highest recovery of phenolics and fucose were observed at 114 µm ultrasound frequency, 0.03 M HCl solvent concentration and 25 min extraction time.

(1) $$\mathrm{Extraction}\;\mathrm{efficiency}\;\left(\%\right)=\frac{\left(V_{t}-V_{c}\right)}{V_{c}}\times 100$$

where V_t_ = values of TPC or TPhC obtained after US treatment and V_c_ = values of corresponding controls of TPC or TPhC.

Ultrasound treatment was found to be more effective in extracting phlorotannin as the acoustic cavitation generated by ultrasound enhanced the release of these compounds from the matrix. It was noted that the duration of treatment affected TPhC in ultrasound treated samples; 30 min resulted in extraction of higher TPhC values in all treated and control samples compared to 10 min, except for UAE conditions (130 kHz, 30 min and 70% ethanol) which resulted in lower TPhC values. Ultrasound treatments showed better extraction yields compared to control in all cases. It was also observed that the extraction time influenced the amount of TPC and TPhC recovered. 

While testing the residues of the control and ultrasound treated samples, the opposite trend was observed. The TPC values in the dried treated residue samples ranged from 125.2 to 218.1 mg GAE/g while TPhC values ranged from 97.4 to 144.6 mg PGE/g. Likewise, TPC and TPhC values in the dried control residue samples ranged from 232.7 to 272.8 mg GAE /g and 125.4 to 179.9 mg PGE/g, respectively. TPC and TPhC values were higher in all extracts compared to residues, indicating that the solvents enabled extraction of most of the phenolics and phlorotannins from the seaweed samples. Samples that showed the highest (579.7 ± 9.2 mg/g) and the lowest (422.7 ± 4.4 mg/g) TPC in the extracts retained the lowest (125.2 ± 1.1 mg/g) and the highest (218.1 ± 2.8 mg/g) phenolic content in their respective residues. Similarly, samples that showed the highest (471.5 ± 7.5 mg/g) and the lowest (327.2 ± 7.2) TPhC in the extracts retained the lowest (TPhC: 97.4 ± 2.9 mg/g) and the highest (TPhC: 144.7± 2.3 mg/g) phlorotannins in their respective residues. While summing the TPC and TPhC values of respective extracts and residues, total polyphenols and total phlorotannins (total in seaweed) varied significantly (*p* < 0.05) in control as well as treated samples. Total polyphenols (extract + residue) ranged from 573.6 ± 6.8 to 591.3 ± 9.2 mg/g in control samples, and ranged from 607.4 ± 9.1 to 704.9 ± 9.6 mg/g of dried extract in treated samples. Similarly, total phlorotannins (extract + residue) in control samples ranged from 382.1 ± 7.5 to 427.7 ± 7.1 mg/g, and ranged from 456.4 ± 4.4 to 568.9 ± 9.9 mg/g of dried extract (Table 1) in treated samples. Variations in total polyphenols and phlorotannins (extract + residue) content may be caused by the non-specific nature of extraction solvents, ultrasound frequencies and spectrophotometric tests for TPC and TPhC. During extraction, other polar compounds (such as sugars, proteins) may be released along with polyphenols [21] which may be detected and quantified along with TPC and TPhC.

### 2.2. Scanning Electron Microscopic Analysis

**Figure 2:**
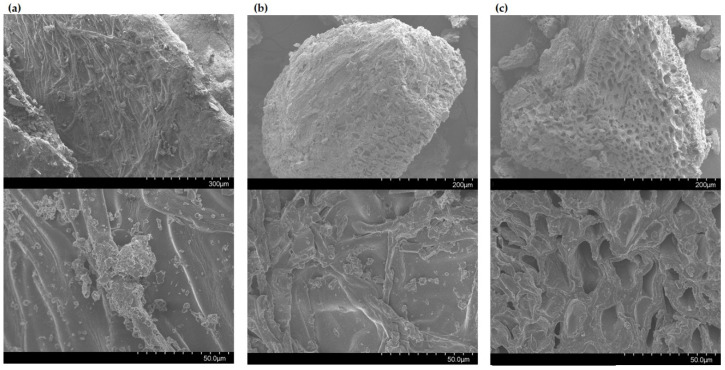
SEM images of *F. vesiculosus* (**a**) untreated samples (intact and dried macroalgae), (**b**) control samples (50% ethanol, 30 min, no ultrasound) and (**c**) UAE treated samples (35 kHz, 50% ethanol, 30 min). Scale bars: 300 µm (magnification 150×), 200 µm (magnification 250×) and 50 µm (magnification: 1000×).

Scanning electron microscopy (SEM) was used to investigate the impact of the extraction treatments on the structure of the treated macroalgal cells. Figure 2 shows the microscopic structure of *F. vesiculosus* biomass prior to extraction (Figure 2a), seaweed residue of control sample (50% ethanol, 30 min, no ultrasound (Figure 2b)) and treated sample after UAE under optimum conditions (35 kHz, 50% ethanol, 30 min (Figure 2c)). It can be observed in the SEM images that the cell surface of the initial seaweed biomass is intact and surrounded by other impurities and residual materials, while the cell surfaces of the control samples (without ultrasound treatment) appear to be smooth with an increased number of pores that allowed diffusion of bioactive compounds to the media. The impact of the optimised UAE conditions (35 kHz, 50% ethanol, 30 min) on the macroalgal biomass are more evident in Figure 2c. The cell surfaces of treated samples exhibit an increased porosity that facilitated the extraction of higher yields of TPC and TPhC compared to control samples (Table 1). Previously, Rodriguez-Jasso et al. [22] and Garcia-Vaquero et al. [23] reported similar findings when evaluating the efficiency of MAE, UAE and UMAE (ultrasound-microwave assisted extraction) to generate extracts from *F. vesiculosus* and *A. nodosum* seaweed.

### 2.3. Comparison of Optimised UAE Conditions and Conventional Solvent Extraction

#### 2.3.1. Extraction Yield and Phenolic Constituents

**Figure 3:**
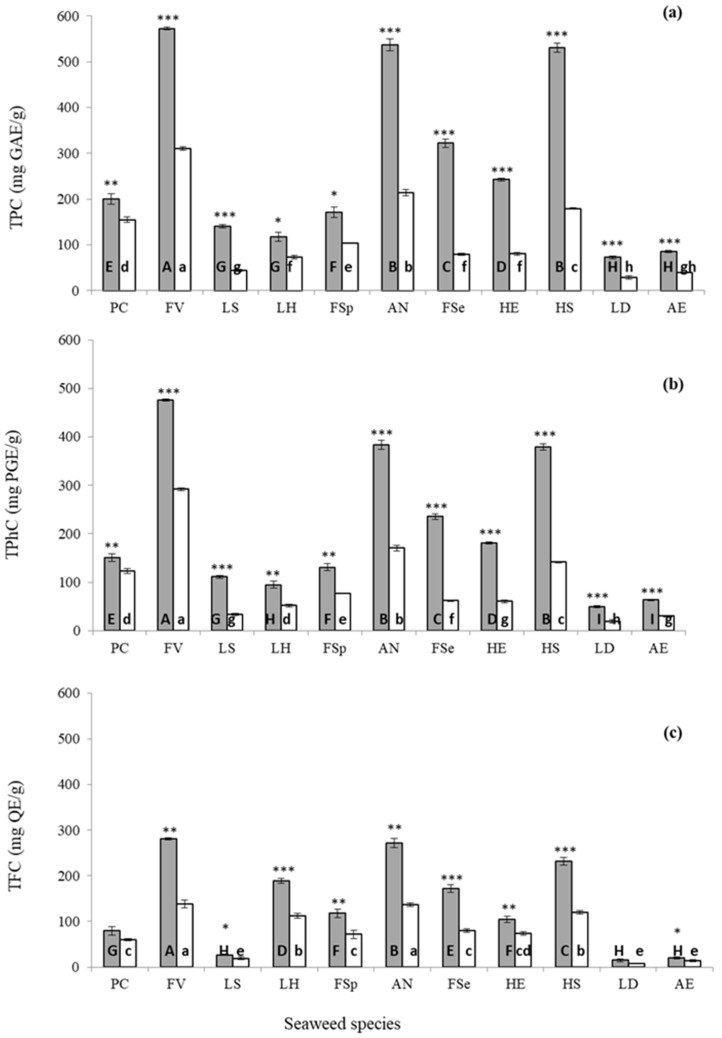
Total polyphenol (**a**), total phlorotannin (**b**) and total flavonoid (**c**) content from 11 seaweed species obtained using UAE (grey bars) and conventional solvent extraction (white bars) technologies. The statistical differences in bioactive compounds extracted using UAE or conventional solvent extraction technologies for each seaweed are represented as * *p* < 0.05, ** *p* < 0.01 and *** *p* < 0.001. Different letters indicate statistical differences (*p* < 0.05) in the yields of bioactive compounds between seaweed obtained by UAE (uppercase letters) or conventional solvent extraction (lowercase letters). TPC (total phenolic content), TPhC (total phlorotannin content) and TFC (total flavonoid content) are expressed as mg gallic acid equivalents (GAE)/g dried weight extract, mg phloroglucinol equivalents (PGE)/g dried weight extract and mg quercetin equivalents (QE)/g dried weight extract, respectively. Abbreviation of seaweed species are as follows: PC (*Pelvetia caniculata*), FV (*Fucus vesiculosus*), LS (*Laminaria saccharina*), LH (*Laminaria hyperborea*), FSp (*Fucus spiralis*), AN (*Ascophyllum nodosum*), FSe (*Fucus serratus*), HE (*Himanthalia elongata*), HS (*Halidrys siliquosa*), LD (*Laminaria digitata*) and AE (*Alaria esculenta*).

UAE efficiency for the extraction of phenolic compounds was further evaluated by applying the optimised UAE conditions (35 kHz, 50% ethanol, 30 min) on 11 seaweed species and comparing the yields obtained to conventional solvent extraction. Extracts recovered from all 11 seaweeds using both UAE and solvent extraction techniques were analysed for extraction yield, TPC, TPhC, total flavonoid content (TFC) and antioxidant capacity (Table 2 and Figure 3). 

UAE extraction yields were statistically (*p* < 0.05) higher than the yields obtained from conventional solvent extraction for all seaweed studied. The yields obtained from conventional extraction were in the range of 10.5%–19.3%, while yields obtained using UAE were in the range of 20.4–36.9% (Table 2). Ultrasound improved the extraction yield 1.5–2.2 fold in all tested seaweeds. Statistical differences were also observed for the yields obtained between different seaweed species even for the same extraction conditions (Table 2). With UAE, the highest extraction yield was obtained from *Laminaria hyperborea* (36.9%), while the lowest yield (20.4%) was obtained from *F. serratus*. Compared to solvent extraction, the highest increase in yield (3.1-fold) using UAE was obtained from *F. vesiculosus,* while the lowest increase in yield (1.5-fold) was obtained from *Pelvetia caniculata*, indicating an extraction yield variation between seaweed species. Extraction yield varies with composition, type and quantity of compounds present in seaweed. Previously, Farvin et al. [24] conducted an extraction study involving 16 seaweeds species using ethanol and water. They observed variation in extraction yields which was attributed to the polarities of compounds present. They also reported that the *F. vesiculosus* extracts obtained using water were more viscous and difficult to extract by passing through a filter, and thus had the lowest yield. Similar findings were reported in a study by Bixler et al. [25] which investigated ultrasound for extraction of phenolic compounds from *Laminaria japonica* using an ionic liquid as solvent. They observed that ultrasound enhanced the extraction yield, by acting as a driving force for dispersing the solvent into the solid samples.

As shown in Figure 3, the levels of TPC, TPhC and TFC extracted using UAE and conventional solvent extraction technologies varied significantly for all 11 seaweeds investigated. UAE enhanced the recovery of bioactive in all 11 seaweeds investigated compared to conventional solvent extraction. The highest recovery of TPC (572.3 ± 3.19 mg GAE/g), TPhC (476.3 ± 2.19 mg PGE/g) and TFC (281.0 ± 1.65 mg QE/g) was recorded from *F. vesiculosus,* while the lowest recovery (TPC: 72.6 ± 2.92 mg GAE/g; TPhC: 50.3 ± 2.01 mg PGE/g; and TFC: 15.2 ± 3.30 mg QE/g) was obtained from *Laminaria digitata* seaweed using optimised UAE conditions. Solvent-led extraction yielded values ranging from 28.7 to 310.1 mg GAE/g for TPC, from 19.0 to 292.0 mg PGE/g for TPhC and from 8.1 to 138.4 mg QE/g for TFC in the 11 seaweeds investigated. The values of TPC (1.3–4.1-fold), TPhC (1.2–3.8-fold) and TFC (1.3–2.1-fold) obtained from UAE treated samples were higher than the values obtained using convention solvent extraction. The highest values of TPC, TPhC and TFC were observed in *F. vesiculosus* while the highest increase of TPC (4.1-fold), TPhC (3.8-fold) and TFC (2.1-fold) was obtained from *Fucus serratus* seaweed. The highest and the lowest values of TPC, TPhC and TFC were recorded in *F. vesiculosus* and *L. digitata* while values of extraction yield recorded the highest and the lowest in *L. hyperborea* and *F. serratus* seaweeds, respectively. Due to the high value of phenolic constituents and extraction yields obtained from *Fucus* species, it can be considered a good source of phenolics compared to other seaweed species investigated. Holdt et al. [2] observed that genus *Fucus* accumulates the highest amount of phlorotannins (up to 12% dry weight). The quantity accumulated depends on the geographical location, season, solar exposure and salinity. 

#### 2.3.2. Antioxidant Capacity Determination

**Figure 4:**
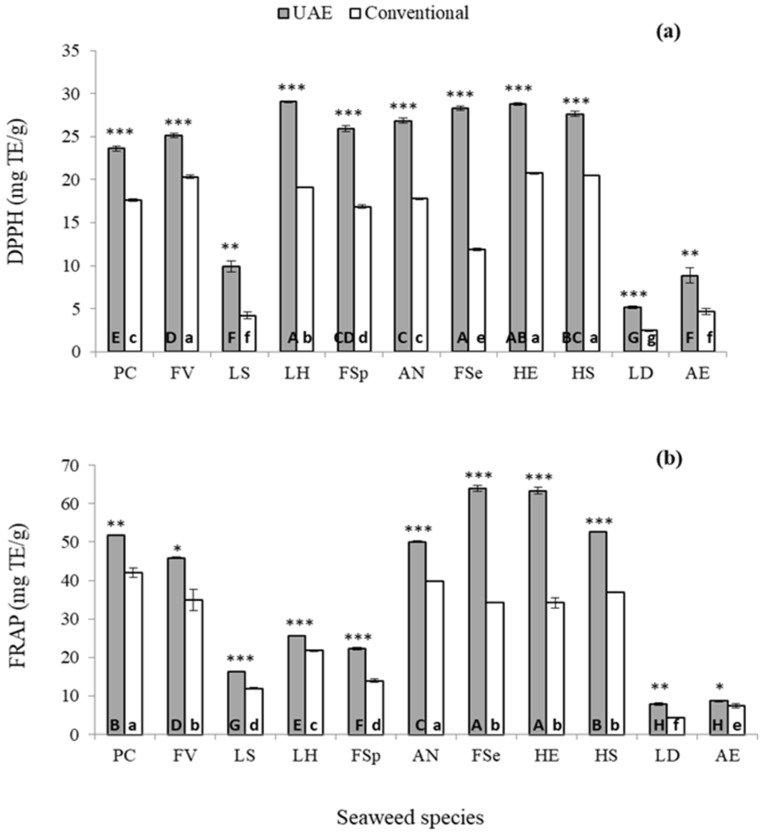
Antioxidant capacity measured as 1,1-diphenyl-2-picryl-hydrazyl (DPPH) activity (**a**) and ferric reducing antioxidant power (FRAP) (**b**) of 11 seaweed extracts obtained from UAE (grey bars) and conventional solvent extraction (white bars) techniques. The statistical differences in antioxidant activity extracted by using UAE or conventional solvent extraction for each seaweed are represented as * *p* < 0.05, ** *p* < 0.01 and *** *p* < 0.001. Different letters indicate statistical differences in the antioxidant activity among seaweed species obtained by UAE (uppercase letters) or conventional solvent extraction (lowercase letters). DPPH and FRAP: expressed as mg trolox equivalent (TE)/g of dry weight extract. Abbreviation of seaweed species are as follow: PC (*Pelvetia caniculata*), FV (*Fucus vesiculosus*), LS (*Laminaria saccharina*), LH (*Laminaria hyperborea*), FSp (*Fucus spiralis*), AN (*Ascophyllum nodosum*), FSe (*Fucus serratus*), HE (*Himanthalia elongata*), HS (*Halidrys siliquosa*), LD (*Laminaria digitata*) and AE (*Alaria esculenta*).

The antioxidant capacity of seaweed extracts recovered from UAE and conventional solvent extraction were analysed using 1,1-diphenyl-2-picryl-hydrazyl (DPPH) and ferric reducing antioxidant power (FRAP) assays (Figure 4). The extract from *F. serratus* obtained by UAE had the strongest DPPH free radical scavenging activity (29.1 ± 0.25 mg TE/g) and the highest FRAP value (63.9 ± 0.74 mg TE/g), while the extract obtained from *L. digitata* had the weakest free radical scavenging activity (5.2 ± 0.15 mg TE/g) and the lowest FRAP value (7.8 ± 0.30 mg TE/g) (Figure 4a). However, for conventional solvent extraction, the extract from *H. elongata* had the strongest DPPH free radical scavenging activity (20.7 ± 0.10 mg TE/g) while the extract from *Pelvetia caniculata* exhibited the highest FRAP value (42.0 ± 1.20 mg TE/g). However, similar to the results observed for UAE, the extract from *L. digitata* obtained by conventional solvent extraction showed the weakest DPPH free radical scavenging activity (2.5 ± 0.09 mg TE/g) and the lowest FRAP value (4.4 ± 0.11 mg TE/g) (Figure 4b). It is likely that that ultrasound facilitated the release of phenolic compounds from the seaweed which exhibited a strong antioxidant capacity. Compared to conventional extraction, UAE increased the DPPH scavenging capacity from 23.6% to 146.4% and reduced the power (FRAP) from 16.6% to 86.7% in the 11 seaweeds investigated. The highest enhancement in DPPH scavenging capacity and FRAP reducing power was observed for *F. serratus* seaweed. However, the lowest increase in DPPH scavenging capacity was recorded for *F. vesiculosus*, while the lowest enhancement in FRAP reducing power was recorded in *Alaria esculenta* seaweed. 

Overall, the extracts recovered from UAE treated seaweeds showed higher antioxidant activity compared to extracts from conventional solvent extraction. Similar results were obtained by Kadam et al. [26], who studied extraction of laminarin from *A. nodosum* and *L. hyperborea*, with conventional solvent extraction and UAE. They reported that the antioxidant activity and total phenolic content were higher in samples treated with ultrasound. The combined ferric reducing/ antioxidant power (FRAP) value of antioxidants in the sample is proportional to the antioxidant potential [27]. A study by Dang et al. [28] reported that when UAE and conventional extraction was performed for extraction of phenolic compounds from *Hormosira banksii* algae, UAE was more effective than conventional solvent extraction to TPC and antioxidant capacity in terms of ABTS, DPPH and FRAP. They reported that ABTS, DPPH and FRAP values using UAE were higher (166.8%, 154.6% and 150.6%, respectively) compared to conventional extraction techniques employed. They also reported that the TPC levels in UAE samples were 142.6% higher than conventionally extracted samples, indicating that ultrasound is effective for extraction of bioactive compounds, as well as giving a good quality extract.

## 3. Materials and Methods

### 3.1. Chemicals

Vanillin, ethanol, methanol, ferric chloride, aluminum chloride, sodium nitrite, sodium carbonate and sodium hydroxide chemicals were purchased from Fisher Scientific (Loughborough, UK). Reagents and standards including 6-hydroxy-2,5,7,8-tetramethylchromane-2-carboxylic acid (trolox), 1,1-diphenyl-2-picryl-hydrazyl (DPPH), 2,4,6-tripyridyl-s-triazine (TPTZ), Folin-Ciocalteau reagent, gallic acid, phloroglucinol, quercetin and catechin were purchased from Sigma-Aldrich Chemical Co. (Steinheim, Germany). All other chemicals used were of analytical grade and were purchased from Sigma-Aldrich.

### 3.2. Seaweed Biomass

The 11 seaweeds used in this study were Fucus serratus, Fucus vesiculosus, Fucus spiralis, Himanthalia elongata, Halidrys siliquosa, Laminaria digitata, Laminaria saccharina, Laminaria hyperborea, Ascophyllum nodosum, Alaria esculenta and Pelvetia caniculata. All seaweeds were harvested (Quality Sea Veg., Co. Donegal, Ireland) and washed to remove any debris and epiphytes attached. Seaweed samples were dried using an air circulating oven following industry practices (50 °C, 9 days), and milled to 1 mm particle size using a hammer mill (Christy and Norris, Chelmsford, UK). The samples were stored at room temperature in dark conditions prior to use.

### 3.3. Ultrasound assisted Extraction (UAE) Procedures

*F. vesiculosus* seaweed was selected for optimisation of UAE conditions to recover high yield of phenolic compounds with antioxidant properties. Dried and milled seaweed samples were mixed with aqueous ethanolic solutions (30%, 50% and 70% v/v) at a ratio of 1:10 (w/v) for phlorotannins and phenolics extraction. The UAE treatments were performed using an ultrasonic water bath (Fisher Bioblock Scientific, Pittsburgh, PA, USA) at 35 and 130 kHz for 10 and 30 min. Control samples were also treated following the same procedures while omitting the use of ultrasound. All the extraction procedures were performed in duplicate. After the treatment, control and ultrasound treated samples were centrifuged at 3000× g for 15 min at 20 °C. The supernatants and residues (pellet) were collected separately and ethanol was evaporated under vacuum. The remaining aqueous fraction of supernatants and residues were freeze-dried and stored at −20°C prior to subsequent analyses.

The most effective optimised UAE conditions (35 kHz, 30 min, 50% ethanol) were further tested on the other 10 seaweed species and the efficiency of UAE was compared to conventional solvent extraction.

### 3.4. Conventional Solvent Extraction

Solvent extraction was carried out on all 11 seaweed species to compare the efficiency of conventional solvent extraction with the optimised UAE conditions determined in this study. Seaweed samples were extracted following the method described by Li et al. [29] with minor modifications. Briefly, samples were mixed with 50% ethanol (1:15, w/v) and extracted in a shaking water bath (20 °C, 200 rpm and 4 h). The supernatant was filtered through Whatman #1 filter paper (Whatman International Limited, Maidstone, UK). The macroalgal pellets were re-extracted following the same procedure, and both supernatants were pooled together. Ethanol was evaporated under vacuum and the remaining aqueous fraction was freeze-dried and stored at −20 °C for further analyses.

### 3.5. Scanning Electron Microscopy

Scanning Electron Microscopy (SEM) was used to investigate the effect of ultrasound on *F. vesiculosus* surface characteristics. Dried seaweed samples and pellets from the extraction conditions achieving high extraction yields (35 kHz, 50% ethanol, 30 min) and control (no ultrasound, 50% ethanol, 30 min) were collected and prepared as described by Garcia-Vaquero et al. [23]. The images were recorded using a SEM Regulus 8230 (Hitachi Ltd., Tokyo, Japan).

### 3.6. Phenolic Composition and Antioxidant Capacity Analysis

All the extracts were analysed for total phenolic content (TPC), total phlorotannin content (TPhC), total flavonoid content (TFC) and antioxidant capacity using 1,1-diphenyl-2-picryl-hydrazyl (DPPH) activity and ferric reducing antioxidant power (FRAP) assays.

#### 3.6.1. Total Phenolic Content (TPC) and Total Phlorotannin Content (TPhC)

The amount of TPC and TPhC in the extracts was determined as outlined by Rajauria et al. [7]. Briefly, 100 μL of sample or standards (gallic acid and phloroglucinol for TPC and TPhC, respectively) was mixed with 2 mL of 2% Na_2_CO_3_, and left to stand for 2 min before adding 100 μL of Folin-Ciocalteau reagent (1:1, v/v). The solutions were mixed and incubated for 30 mins at room temperature in dark conditions. The absorbance of the reaction was read at 720 nm using a spectrophotometer (UVmini-1240, Shimadzu, Kyoto, Japan). All the measurements were done in triplicate. The TPC was expressed as mg gallic acid equivalent per gram (mg GAE/g) dried extract and the TPhC was expressed as mg phloroglucinol equivalents per gram (mg PGE/g) dried extract. 

#### 3.6.2. Total Flavonoid Content

The total flavonoid content (TFC) was determined using the method described by [30] with slight modifications. Briefly, 250 μL of each extract or standard solution was mixed with 1.475 mL distilled water and 75 μL sodium nitrite (5%) solution followed by the addition of 150 μL aluminum chloride hexahydrate (10%) after 6 min, and then mixed. After 5 min, 0.5 mL of sodium hydroxide (1 M) solution was added to the reaction mixture and the absorbance against blank was determined at 510 nm. Quercetin was used as a reference compound and the results were expressed as mg quercetin equivalents per gram (mg QE/g) dried extract.

#### 3.6.3. DPPH Radical-scavenging Assay

The DPPH free radical scavenging activity was conducted as per the method reported by Sridhar and Charles [31]. Briefly, 700 µL of sample or standard was mixed well with 700 µL of 100 µM DPPH methanolic solution in a test tube. The reaction mixture was incubated at room temperature in the dark for 20 min and read against a blank of methanol (without DPPH solution) at 515 nm using a UV-Vis spectrophotometer. Samples were prepared in triplicate. Trolox standard was used to generate a standard curve and results were expressed as mg trolox equivalents (TE)/g dry weight extract. The inhibition percentage of scavenging of DPPH was calculated using Equation (2).

(2) $$\mathrm{DPPH}\;\mathrm{radical}\;\mathrm{scavenging}\;\mathrm{capacity}\;(\%)=\frac{\left(A_{\mathrm{control}}{\;-\;A}_{\mathrm{sample}}\right)}{A_{\mathrm{control}}}\times 100$$

where “A_control_” is the absorbance of the control (DPPH solution without sample/standard), “A_sample_” is the absorbance of the test sample (DPPH solution plus test sample/standard).

#### 3.6.4. Ferric Reducing Antioxidant Power (FRAP) Assay

The total antioxidant reducing power of seaweed extracts and standard was measured using the ferric reducing antioxidant power (FRAP) assay as reported by Benzie and Strain [32]. Preheated 2.5 mL FRAP reagent (300 mM acetate buffer, pH 3.6; 10 mM 2,4,6-Tri(2-pyridyl)-s-triazine in 40 mM hydrochloric acid and 20 mM Iron(III) chloride hexahydrate in the ratio of 10:1:1, *v/v/v*) at 37 °C was mixed with 83 µL of samples or standard and incubated in dark at room temperature for 10 min. Trolox was used as a standard and the absorbance of the standard or samples was recorded at 593 nm against a reagent blank containing FRAP reagent only. The results were expressed as mg trolox equivalents (TE)/g dry weight extract.

### 3.7. Statistical Analysis

All the experiments were carried out in triplicate. Results are expressed as mean ± standard deviation. Statistical analysis was performed using SPSS version 24.0 (IBM, Armonk, NY, USA). The effects of ultrasound frequency, extraction time and solvents on the recovery of total polyphenols, total phlorotannins and associated antioxidant activities were analysed using ANOVA and the differences analysed further by Student’s *t*-tests and Tukey’s HSD post-hoc tests. In all cases, differences were considered statistically significant at *p* < 0.05. 

## 4. Conclusions

UAE was found to be more effective than conventional solvent extraction for extraction of bioactive compounds compared. It was observed that ethanol concentration, ultrasound frequency and duration of extraction influenced extraction yield and the phenolic compounds obtained. The optimised UAE treatment based on extraction yield, total phenol and total phlorotannin content recovered was found to be 35 kHz for 30 min with 50% ethanol. UAE resulted in higher extraction yield of extracts and higher values of TPC, TPhC and TFC and antioxidant capacities for all 11 seaweeds studied compared to conventional solvent extraction. It was also noted that the response to the extraction technique was species specific. The TPC obtained using UAE was highest for *F. vesiculosus* but the largest enhancement of extraction (4.1-fold) was achieved for *F. serratus* seaweed. Likewise, despite the highest extract yield and polyphenolic concentration observed for *F. vesiculosus*, the highest antioxidant capacity was recorded for *F. serratus*, using UAE, which indicates that there is no direct relationship between phenolic content and antioxidant activities. It is recommended that a full profiling of phenolic compounds in the extract should be carried out prior to its utilisation for commercial applications.

## Figures and Tables

**Table 1:** Effect of ultrasound assisted extraction process parameters (time, ultrasound frequencies and solvents) on total phenolic content (TPC) and total phlorotannin content (TPhC) of *F. vesiculosus* seaweed.

Extraction Solvent	US Frequencies	Extraction Time (min)	TPC (Extract) (mg GAE/g)	TPC (Residue) (mg GAE/g)	TPC (Total) (mg GAE/g)	TPhC (Extract) (mg PGE/g)	TPhC (Residue) (mg PGE/g)	TPhC (Total) (mg PGE/g)
30% ethanol	Control *	10	314.5 ± 5.9 **c**	262.9 ± 2.8 **a**	577.5 ± 8.7 **c**	222.0 ± 4.8 **c**	179.9 ± 2.8 **a**	401.9 ± 7.6 **c**
		30	340.3 ± 9.4 **C**	247.8 ± 1.4 **A**	588.1 ± 4.9 **C**	248.7 ± 5.9 **C**	178.9 ± 1.2 **A**	427.7 ± 7.1 **C**
	35 kHz	10	422.7 ± 4.4 **b**	218.1 ± 2.8 **b**	640.9 ± 7.2 **b**	327.2 ± 7.2 **b**	144.7 ± 2.3 **b**	471.9 ± 9.5 **a**
		30	463.7 ± 5.3 **B**	201.2 ± 3.2 **B**	664.9 ± 8.5 **B**	392.3 ± 5.5 **B**	122.1 ± 2.6 **B**	514.4 ± 8.2 **B**
	130 kHz	10	453.9 ± 6.7 **a**	190.9 ± 2.9 **c**	644.9 ± 9.7 **a**	347.8 ± 4.4 **a**	116.1 ± 2.4 **c**	463.9 ± 6.8 **b**
		30	579.7 ± 9.2 **A**	125.2±0.4 **C**	704.9 ± 9.6 **A**	471.5 ± 7.5 **A**	97.4 ± 2.9 **C**	568.9 ± 9.9 **A**
50% ethanol	Control *	10	338.0 ± 4.9 **b**	235.6 ± 1.8 **a**	573.6 ± 6.8 **b**	262.2 ± 4.0 **c**	144.1 ± 1.5 **a**	406.2 ± 5.6 **c**
		30	358.5 ± 5.3 **C**	232.7 ± 3.9 **A**	591.3 ± 9.2 **C**	286.2 ± 4.4 **C**	125.4 ± 3.2 **A**	411.6 ± 7.6 **C**
	35 kHz	10	464.7 ± 9.8 **a**	179.6 ± 3.5 **c**	644.3 ± 13.4 **a**	408.7 ± 4.1 **a**	121.7 ± 2.9 **b**	530.4 ± 7.0 **a**
		30	571.1 ± 10.0 **A**	125.6 ± 1.1 **C**	696.7 ± 11.7 **A**	462.6 ± 2.1 **A**	99.8 ± 2.9 **B**	562.4 ± 5.1 **A**
	130 kHz	10	458.6 ± 10.8 **a**	190.0 ± 1.9 **b**	648.7 ± 12.7 **a**	350.1 ± 6.9 **b**	144.6 ± 1.6 **a**	494.8 ± 8.5 **b**
		30	474.1 ± 12.7 **B**	180.6 ± 1.1 **B**	654.7 ± 13.9 **B**	366.5 ± 4.0 **B**	119.8 ± 1.9 **A**	486.3 ± 6.0 **B**
70% ethanol	Control *	10	306.8 ± 7.3 **c**	272.8 ± 3.2 a	579.6 ± 10.5 c	234.8 ± 5.0 **c**	147.3 ± 2.5 **a**	382.1 ± 7.5 **b**
		30	316.4 ± 6.1 **C**	271.8 ± 0.9 A	588.2 ± 6.9 C	253.6 ± 5.0 **C**	153.4 ± 1.7 **A**	406.9 ± 6.7 **C**
	35 kHz	10	436.9 ± 7.3 **b**	195.6 ± 3.7 b	632.5 ± 10.9 a	362.4 ± 5.3 **a**	119.5 ± 3.0 **b**	481.9 ± 8.3 **a**
		30	503.2 ± 9.2 **A**	158.8 ± 3.3 B	662.0 ± 12.5 A	389.9 ± 3.5 **A**	106.4 ± 2.9 **C**	496.3 ± 6.4 **A**
	130 kHz	10	453.2 ± 6.8 **a**	154.2 ± 2.2 c	607.4 ± 9.1 b	347.2 ± 2.2 **b**	122.7 ± 6.7 **b**	469.9 ± 8.9 **a**
		30	468.1 ± 10.4 **B**	149.2 ± 2.6 B	617.3 ± 13.0 B	334.8 ± 2.2 **B**	121.7 ± 2.1 **B**	456.4 ± 4.4 **B**

* Control: no ultrasound. Results are expressed as average ± standard deviation (n = 3); different letters indicate statistical differences (*p* < 0.05) on the recovery of compounds by applying either 10 (**a**–**c**) or 30 min (**A**–**C**) within the same extraction treatment conditions; TPC (total phenolic content) and TPhC (total phlorotannin content) are expressed as mg gallic acid equivalents (GAE)/g dried weight extract and mg phloroglucinol equivalents (PGE)/g dried weight extract, respectively.

**Table 2:** Extraction yield (%) obtained from selected seaweed species using UAE and conventional solvent extraction techniques.

Seaweed Species	Extraction Yield (%)	
	UAE	Conventional
*Pelvetia caniculata*	20.5 ± 0.35 **h**	14.0 ± 0.28 ***e***
*Fucus vesiculosus*	35.1 ± 0.33 **b**	11.2 ± 0.29 ***g***
*Laminaria saccharina*	30.9 ± 0.41 **d**	17.0 ± 0.47 ***c***
*Laminaria hyperborea*	36.9 ± 0.11 **a**	19.3 ± 0.21 ***a***
*Fucus spiralis*	25.2 ± 0.50 **f**	14.7 ± 0.45 ***de***
*Ascophyllum nodosum*	24.4 ± 0.31 **f**	12.7 ± 0.15 ***f***
*Fucus serratus*	20.4 ± 0.19 **h**	10.5 ± 0.12 ***g***
*Himanthalia elongata*	23.4 ± 0.30 **g**	15.0 ± 0.26 ***d***
*Halidrys siliquosa*	29.0 ± 0.25 **e**	14.5 ± 0.31 ***de***
*Laminaria digitata*	29.4 ± 0.16 **e**	18.4 ± 0.21 ***b***
*Alaria esculenta*	33.0 ± 0.06 **c**	17.2 ± 0.09 ***c***

Results are expressed as average ± standard deviation (n = 6). Different letters indicate statistical differences (*p* < 0.05) between the yield of extracts obtained from multiple seaweed species by using UAE (lowercase letters) and conventional extraction conditions (italic letters). The extraction yield is calculated by using following formula: extraction yield (%) = (weight of dry extract / weight of dry sample) × 100.

## Supplementary Materials

Supplementary File 1
