# Supplementary material for: Optimisation of Ultrasound Frequency, Extraction Time and Solvent for the Recovery of Polyphenols, Phlorotannins and Associated Antioxidant Activity from Brown Seaweeds
Source: Mar Drugs. 2020 May 11;18(5):250. doi: 10.3390/md18050250 (PMC7281582; doi:10.3390/md18050250)
Supplement: Supplementary File 1 [file marinedrugs-18-00250-s001.pdf]

Supplementary Data

Figure 3. Total polyphenol (a), total phlorotannin (b) and total flavonoid (c) content from 11 seaweed species obtained using UAE and conventional solvent extraction technologies.

Seaweed species	TPC (mg GAE/g)		TPhC (mg PGE/g)		TFC (mg QE/g)	
	UAE	Conventional	UAE	Conventional	UAE	Conventional
Pelvetia caniculata	199.6±11.4 E	154.8±6.5 d	151.3±7.9 E	123.0±5.3 d	79.4±8.7 G	59.3±2.3 c
Fucus vesiculosus	572.3±3.2 A	310.1±3.5 a	476.3±2.2 A	292.0±2.8 a	281.0±1.6 A	138.4±8.7 a
Laminaria saccharina	140.0±4.0 G	44.2±2.1 g	111.1±2.7 G	33.8±1.7 g	26.7±1.6 H	18.9±3.1 e
Laminaria hyperborea	117.5±10.3 G	73.4±3.4 f	94.8±7.1 H	52.2±2.8 d	188.6±5.7 D	112.4±5.3 b
Fucus spiralis	170.5±11.5 F	104.3±0.8 e	131.3±7.9 F	77.3±0.7 e	118.1±9.2 F	71.5±9.6 c
Ascophyllum nodosum	536.9±13.5 B	213.8±7.0 b	383.2±9.3 B	170.8±5.7 b	271.4±9.9 B	136.7±3.8 a
Fucus serratus	322.0±8.6 C	79.4±2.2 f	235.5±5.9 C	62.0±1.8 f	171.4±8.3 E	79.8±4.2 c
Himanthalia elongata	242.6±3.3 D	80.3±2.9 f	180.9±2.3 D	60.7±2.3 g	104.8±7.2 F	73.2±3.9 cd
Halidrys siliquosa	530.8±9.5 B	179.1±1.6 c	379.0±6.5 B	141.9±1.3 c	231.4±8.2 C	120.1±4.1 b
Laminaria digitata	72.6±2.9 H	28.7±4.0 h	50.3±2.0 I	19.0±3.3 h	15.2±3.3 H	8.1±0.9 e
Alaria esculenta	84.6±2.3 H	39.3±1.4 gh	63.8±1.6 I	30.2±1.2 g	20.0±2.9 H	13.7±1.8 e

The statistical differences in bioactive compounds extracted using UAE (ultrasound-assisted extraction) or conventional solvent extraction technologies for each seaweed are represented as * $p < 0.05$, ** $p < 0.01$ and *** $p < 0.001$. Different letters indicate statistical differences ($p < 0.05$) in the yields of bioactive compounds between seaweed obtained by UAE (uppercase letters) or conventional solvent extraction (lowercase letters). TPC (total phenolic content), TPhC (total phlorotannin content) and TFC (total flavonoid content) are expressed as mg gallic acid equivalents (GAE)/g dried weight extract, mg phloroglucinol equivalents (PGE)/g dried weight extract and mg quercetin equivalents (QE)/g dried weight extract, respectively.

Figure 4. Antioxidant capacity measured as DPPH (**a**) and FRAP (**b**) of 11 seaweed extracts obtained from UAE and conventional solvent extraction techniques.

Seaweed species	DPPH (mg TE/g)		FRAP (mg TE/g)	
	UAE	Conventional	UAE	Conventional
Pelvetia caniculata	23.63±0.32 E	17.64±0.14 c	51.69±0.15 B	42.04±1.20 a
Fucus vesiculosus	25.09±0.25 D	20.30±0.21 a	45.87±0.20 D	34.82±2.79 b
Laminaria saccharina	9.90±0.65 F	4.20±0.40 f	16.34±0.10 G	11.92±0.19 d
Laminaria hyperborea	29.07±0.09 A	19.11±0.08 b	25.61±0.18 E	21.78±0.27 c
Fucus spiralis	25.92±0.32 CD	16.87±0.16 d	22.27±0.34 F	13.97±0.39 d
Ascophyllum nodosum	26.86±0.32 C	17.80±0.01 c	50.07±0.17 C	39.89±0.08 a
Fucus serratus	29.28±0.25 A	11.88±0.10 e	63.91±0.74 A	34.23±0.20 b
Himanthalia elongata	28.77±0.17 AB	20.74±0.10 a	63.29±0.91 A	34.21±1.33 b
Halidrys siliquosa	27.64±0.28 BC	20.46±0.03 a	52.65±0.18 B	36.83±0.14 b
Laminaria digitata	5.21±0.15 G	2.46±0.09 g	7.83±0.30 H	4.36±0.11 f
Alaria esculenta	8.86±0.87 F	4.70±0.36 f	8.66±0.02 H	7.43±0.47 e

The statistical differences in antioxidant activity extracted by using UAE (ultrasound-assisted extraction) or conventional solvent extraction for each seaweed are represented as * $p < 0.05$, ** $p < 0.01$ and *** $p < 0.001$. Different letters indicate statistical differences in the antioxidant activity among seaweed species obtained by UAE (uppercase letters) or conventional solvent extraction (lowercase letters). DPPH and FRAP: expressed as mg trolox equivalent (TE)/g of dry weight extract.
